# Undiagnosed Uterine Didelphys, Concomitant Right Renal Agenesis, and Left Nephrolithiasis in a Primigravida With Breech Pregnancy: A Case Report

**DOI:** 10.7759/cureus.65573

**Published:** 2024-07-28

**Authors:** Amyny Aisha Che Musa, Mohd Hafizuddin Husin, Mohd Ezane Aziz, Mohd Azaad A. Hamid, Erinna Mohamad Zon

**Affiliations:** 1 Department of Radiology, School of Medical Sciences, Health Campus, Universiti Sains Malaysia, Kelantan, MYS; 2 Department of Obstetrics and Gynaecology, School of Medical Sciences, Health Campus, Universiti Sains Malaysia, Kelantan, MYS

**Keywords:** ecv, external cephalic version, breech pregnancy, renal agenesis, uterine didelphys, mullerian anomalies

## Abstract

Mullerian duct anomalies are congenital abnormalities involving the female genital systems. A double uterus or uterine didelphys is one of the uterine duplication anomalies that result from impaired fusion with regard to the Mullerian ducts. The uterine didelphys can be diagnosed earlier in symptomatic patients. However, the diagnosis can be delayed or the patients may remain undiagnosed throughout their lifetime if asymptomatic. Pregnant women with uterine didelphys are at a greater risk for spontaneous miscarriage, malposition, premature labor, and cervical incompetence. Uterine didelphys are also associated with renal anomalies such as renal agenesis or duplex kidneys.

We present a case of a 24-year-old primigravida who had a spontaneous pregnancy and underwent a lower segment cesarean section (LSCS) after a failed external cephalic version for the breech fetal position. The uterine didelphys was diagnosed postnatally. Her condition was associated with right renal agenesis and concomitant left renal calculus.

## Introduction

Mullerian duct anomalies refer to congenital defects in the female genital system due to abnormal embryological development. This abnormal development can result in complete agenesis, defective fusion, or failed resorption. According to Pfeifer et al., these conditions can be classified into nine subtypes: complex anomalies, transverse vaginal septum, longitudinal vaginal septum, septate uterus, bicornuate uterus, uterus didelphys, unicornuate uterus, cervical agenesis, and Mullerian agenesis [1]. According to Shilpa, uterine didelphys has a better prognosis in terms of pregnancy than other uterine anomalies due to the improved blood supply and collateral connections between the two horns [2]. As per Chan et al., the pregnancy outcome in a uterine didelphic patient showed that the conception rate is 0.9, with the first and second-trimester abortion rates of 1.1 and 1.39, respectively. The rate of preterm birth risk is increased, which is about 3.58 times higher, with the rate of malpresentation being approximately 3.35 [3].

There is an association between uterine and renal anomalies due to the close relation of embryological development between these urinary and reproductive organs. Thus, renal tract abnormality can be seen and should be anticipated in patients with Mullerian duct pathology. Heinonen has stated that about 17.3% of women having uterine anomalies suffer from associated renal tract malformations, the most prevalent being patients with the unicornuate uterus (about 29.5%), followed by the didelphic uterus (29.1%). Of note, the most prevalent renal anomaly is unilateral renal agenesis (11.2%), which accounts for 64.6% of all renal tract anomalies [4]. A rare variant of Mullerian duct anomalies is Herlyn-Werner-Wunderlich Syndrome, also called obstructed hemivagina and ipsilateral renal anomaly (OHVIRA). It constitutes a triad of uterus didelphys, unilateral obstructed hemivagina, and ipsilateral renal agenesis.

## Case presentation

A 24-year-old Vietnamese primigravida presented to the obstetric clinic at 37 weeks of gestation for consultation due to persistent breech presentation, diagnosed at 28 weeks of gestational age. She had so far had an uneventful pregnancy. During the visit, she was counseled and decided to undergo an external cephalic version (ECV) procedure. ECV was attempted at 37 weeks of gestational age; however, the procedure failed. Correspondingly, she was electively admitted for a lower segment cesarean section (LSCS). On admission at 38 weeks of gestation, she successfully underwent LSCS and delivered a healthy baby girl weighing 2.5 kg. Intraoperatively, the uterus was didelphic, with the pregnancy on the left uterine horn. There were two uterine cervices and an absence of a vaginal septum. Bilateral fallopian tubes and ovaries were normal.

**Figure 1:**
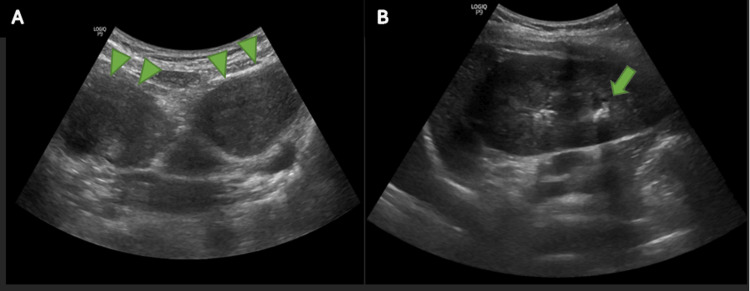
Ultrasonography images (A) Ultrasonography image in the axial view showing abnormal uterine shape with double horn uterus (green arrowheads) in keeping with uterine didelphys. (B) Ultrasonography of the left kidney at the left renal fossa in the longitudinal view illustrates a focus with posterior shadowing in keeping with a calculus (green arrow)

**Figure 2:**
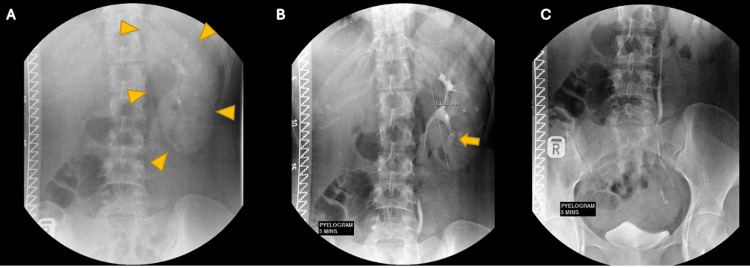
IVU findings IVU in the immediate nephrogenic film (A) shows an absence of the right kidney nephrogram with a normal left renal nephrogram (yellow arrowheads). (B-C) Five-minute pyelographic films show the absence of the right renal collecting system. There is a filling defect in the left lower pole collecting system consistent with the renal calculus, as seen in the ultrasound (yellow arrow) IVU: intravenous urogram

A transabdominal ultrasound was performed postnatally to look for renal abnormalities. A stone in the left renal lower pole and a uterine didelphys were observed (Figure 1). The right kidney was absent in the right renal fossa. An intravenous urogram (IVU) was performed and confirmed the absence of the right kidney with a filling defect in the lower pole of the left kidney consistent with the renal calculus (Figure 2).

The patient was properly counseled regarding her current condition and advised about maintaining pregnancy intervals of two to three years. On further questioning, the patient denied any recurrent urinary tract infection symptoms, hematuria, or renal colic. She was then referred to the Urology team and, subsequently, regular renal function follow-up has been planned for her left renal calculus, and early intervention is anticipated.

## Discussion

Uterine didelphys is a type of uterine duplication anomaly caused by complete failure regarding the fusion of Mullerian ducts, resulting in two corpora of the uterus and two cervix. It accounts for 11.1% of uterine anomalies [5]. Most of the patients with uterine didelphys have some reproductive or gestational problems. However, some may present with dysmenorrhea, coital pain, continuous bleeding despite tampon placement, and difficulties inserting tampons due to the presence of the longitudinal vaginal septum [2,6,7]. Most healthy and asymptomatic Mullerian duct anomaly patients are diagnosed incidentally for renal tract abnormalities during routine imaging. In early pregnancy, transvaginal ultrasonography is a suitable diagnostic method for detecting uterine anomalies. A small portion of these patients can have viable pregnancies, and uterine didelphys has a better pregnancy prognosis than bicornuate, septate, or arcuate uteri [6].

In most cases, congenital uterine abnormalities are associated with malpresentation or preterm delivery. Uterine anomaly is listed as one of the contraindications for ECV [8]. Unaware of the presence of a uterine anomaly, an ECV was attempted on our patient. In patients with uterus didelphys, malposition such as breech presentation and nonpregnant uterus possibly blocking the pelvic inlet and subsequently causing dystocia are two significant factors for a higher rate of cesarean sections. In one study, about 17.3% of 376 uterine anomalies were associated with renal tract abnormality [9].

The most typical abnormality is unilateral renal agenesis (11.2%) and mainly accompanies the vaginal or cervix anomalies seen in uterine didelphys, complete bicornuate, unicornuate, septate uterus, or Mullerian agenesis [4]. OHVIRA syndrome is a rarer Mullerian duct abnormality, and it consists of three components: uterine didelphys, obstructed hemivagina, as well as renal agenesis. Of note, this condition may also be associated with other renal anomalies, such as dysplastic kidney and renal duplication [10]. It should be included in the differential diagnoses in a patient with persistent vaginal discharge, pelvic pain and mass, urinary problems, and primary fertility issues [11].

Although congenital renal abnormalities may remain asymptomatic, their consequences should not be neglected. Early recognition of renal agenesis is essential given the increased risk of renal disease in the remaining single kidney. Patients with a single functional kidney lack spare renal capacity and are more susceptible to kidney-threatening illnesses. These patients also have a higher chance of acquiring hypertension, proteinuria, and glomerulosclerosis, leading to end-stage renal and cardiovascular disease earlier in life. Over 20% of all patients with congenital kidney and urinary system abnormalities will require dialysis by the age of 30 years [9].

Right renal agenesis was diagnosed postnatally in our patient as she had never exhibited any signs and symptoms in her entire life. Unfortunately, there was a calculus present in her solitary left kidney. Nephrolithiasis contributes to about 10% of community-acquired acute kidney injuries as it can cause obstructive nephropathy. The risk for renal failure increases due to direct cellular toxicity caused by the stone’s crystals. It will lead to the damage of tubular epithelial cells and, subsequently, loss of function of the renal parenchyma, especially in cases of recurrent calculi [12].

## Conclusions

Delays in the diagnosis of uterine anomalies during pregnancy and labor may cause uncertainty in monitoring and management, especially in centers without obstetricians or gynecologists. A transabdominal or transvaginal sonogram plays a significant role in detecting uterine abnormalities, especially in the early trimester. Patients with uterus didelphys are considered high-risk and need proper prenatal and antenatal care. Mullerian duct anomalies are also highly associated with congenital renal abnormalities. This case report emphasizes the importance of detecting uterine anomalies in the primary care setting, and the patients should be routinely examined for any coexisting renal and non-communicable diseases, such as diabetes or hypertension. It is critical that the patient is made aware of the problems that may arise later. We believe this report will raise awareness about thoroughly examining all women regarding uterine anomalies to enable an appropriate diagnosis and a timely referral.
